# Do pathogens always evolve to be less virulent? The virulence–transmission trade-off in light of the COVID-19 pandemic

**DOI:** 10.1007/s42977-023-00159-2

**Published:** 2023-03-31

**Authors:** Ádám Kun, András G. Hubai, Adrienn Král, Judit Mokos, Benjámin Áron Mikulecz, Ádám Radványi

**Affiliations:** 1 Present Address: Department of Plant Systematics, Ecology and Theoretical Biology, Institute of Biology, Eötvös University, Pázmány Péter Sétány 1/C, Budapest, 1117 Hungary; 2 Institute of Evolution, Centre for Ecological Research, Konkoly-Thege M. út 29–33, Budapest, 1121 Hungary; 3 Parmenides Centre for the Conceptual Foundation of Science, Parmenides Foundation, Hindenburgstr. 15, 82343 Pöcking, Germany; 4 MTA-ELTE Theoretical Biology and Evolutionary Ecology Research Group, Pázmány Péter Sétány 1/C, Budapest, 1117 Hungary; 5 Alfréd Rényi Institute of Mathematics, Reáltanoda Street 13-15, Budapest, 1053 Hungary; 6 Centre for Data Science and Digital Development, Moholy-Nagy University of Art and Design, Zugligeti út 9–25, Budapest, 1121 Hungary; 7 National Laboratory for Health Security, Eötvös Loránd University, Budapest, Hungary

**Keywords:** COVID-19, SARS-CoV2, Epidemiology, Evolution of virulence, Virulence–transmission trade-off, Law of declining virulence, Avirulence hypothesis

## Abstract

The direction the evolution of virulence takes in connection with any pathogen is a long-standing question. Formerly, it was theorized that pathogens should always evolve to be less virulent. As observations were not in line with this theoretical outcome, new theories emerged, chief among them the transmission–virulence trade-off hypotheses, which predicts an intermediate level of virulence as the endpoint of evolution. At the moment, we are very much interested in the future evolution of COVID-19’s virulence. Here, we show that the disease does not fulfill all the assumptions of the hypothesis. In the case of COVID-19, a higher viral load does not mean a higher risk of death; immunity is not long-lasting; other hosts can act as reservoirs for the virus; and death as a consequence of viral infection does not shorten the infectious period. Consequently, we cannot predict the short- or long-term evolution of the virulence of COVID-19.

## Introduction

During the waves of the COVID-19 pandemic, people often wonder about the future of the virus and its effect on us. One such effect in the spotlight these days is the evolution of virulence, the hereditary change in the relative ability of an infectious agent to cause disease. It is worth mentioning, as reviews on the subject often do, that there are different notions of virulence. Here, we use the definition of decreasing the fitness of the host mostly via increased mortality but also by decreased fecundity (Alizon and Michalakis 2015; Cressler et al. 2015; Geoghegan and Holmes 2018; Read 1994; Sigmund et al. 2002) (In some contexts, virulence can mean the ability to gain entrance to the host or local spreading (infectivity)). When it comes to contemplating the future survival chances of hosts, conventional wisdom is often invoked that a pathogen will become less virulent (less lethal) as it evolves. This expectation stems from two model results and a selective view on current pathogens.

According to the *avirulence hypothesis* put forward by Theobald Smith (Smith 1904), virulence is expected to decrease to zero in the course of evolution. It is not in the interest of a pathogen to harm or kill its host, only to replicate itself. The death of the host terminates the infectious stage of the pathogen, thus ending transmission. Without the excess death caused by the pathogen, the host can be the vehicle for the replication and transmission of the pathogen for a longer time.

The second proposition, often referred to as the *transmission–virulence trade-off*, came as a critique of the *avirulence hypothesis* (Anderson and May 1982; Frank 1996). It acknowledges that the death of the host shortens the time-period in which the pathogen can be transmitted. But it shows that if virulence and transmission are positively correlated but in a decelerating manner, i.e., increasing virulence has a diminishing return in terms of higher transmission, then, there is an intermediate, optimal virulence. Thus, there is an optimal virulence above which it is not evolutionary advantageous for the pathogen to increase virulence. If virulence happens to be higher than this at the beginning (at first contact with the pathogen), it will evolve to lower levels.

The supposed inevitability of lower virulence is a comforting idea during a pandemic. On the other hand, there is an unfounded fear in the general public that pathogens are out there to kill us. And if a pathogen is not deadly enough, it will become more so with every new mutation it acquires. Both of the above theoretical results alleviate this fear, as they agree that no pathogen would evolve to be 100% lethal. Humans tend to recall more salient cases (Phelps and Sharot 2008; Talarico and Rubin 2003), and therefore, one might only remember the very lethal or the very mild pathogens. COVID-19 has symptoms much like the common cold (caused in part by other coronaviruses) or influenza. The common cold is not a deadly disease, and while influenza causes more deaths (CDC 2020, Paget et al. 2019), it is also not a deadly one. This might usher a layperson to jump to the conclusion that COVID-19 will become a common cold-like disease.

In this essay, we shall revisit the avirulence and transmission–virulence hypotheses and discuss the circumstances where their predictions are expected to be true. In applying theoretical results, it is key that underlying assumptions are understood. These assumptions will be compared with what we know about COVID-19 in order to see if they fit. We should be sure to base our hopes and policies on theories that apply—to this particular pathogen (SARS-CoV2 (Gorbalenya et al. 2020)) and the disease it causes (COVID-19)—otherwise we may cause more harm than good.

## “The law of declining virulence”

Around the beginning of the twentieth century, a view of virulence evolution emerged, which held sway for most of that century (Méthot 2012): pathogens gain little by harming their host and thus, should evolve to be non-virulent. The formulation of the hypothesis is often credited to Theobald Smith (Smith 1904). The original formulation and much of its use were verbal, but it can be formulated mathematically (Anderson and May 1982). Let us do so by relying on the SIR model of epidemiology (Kermack et al. 1927) (Eqs. 1–3), where the population is divided into Susceptible, Infectious, and Recovered individuals:

1 $$\frac{{{\text{d}}S}}{{{\text{d}}x}} = - \beta {SI,}$$

2 $$\frac{{{\text{d}}I}}{{{\text{d}}x}} = \beta SI - \gamma I - \delta I,$$

3 $$\frac{{{\text{d}}R}}{{{\text{d}}x}} = \gamma I,$$

where *β* is the transmission rate, *γ* is the recovery rate (1/day), and *δ* is the excess death rate caused by the infection. The basic reproduction number, the number of secondary infections an infected individual produces on average in a susceptible population, is:

4 $$R_{0} \sim \;\frac{\beta }{{\gamma + \delta }}.$$

If the above three parameters are independent of each other, then increasing the transmission rate (*β*), and decreasing the recovery (*γ*) or death rate (*δ*) would increase the basic reproduction number and thus fitness. The transmission rate (*β*) can increase but not indefinitely: Both the speed of infection cannot be instantaneous, and the number of susceptible hosts within reach cannot be unlimited (i.e., pathogens cannot teleport from one host to another). Recovery rate (*γ*) can also decrease, meaning it takes longer and longer to eliminate the pathogen. In the end, a persistent productive infection (*γ* = 0) is possible. Pathogen-caused excess death rate (*δ*) could also decrease and can theoretically reach zero. This evolutionary outcome is avirulence. The outcome heavily rests on the assumption of independence of the parameters.

## The transmission–virulence trade-off

The transmission–virulence trade-off hypothesis posits that from an evolutionary point of view, it is not beneficial for the pathogen to kill its host before being passed to another host. In this regard, it is similar to the avirulence hypothesis. However, the replication of the pathogen within the host causes some inevitable damage to the host. Thus, an increase in the number of pathogens increases both transmission and virulence. Both replication and transmission require a living and, in many cases, active host. If the host is dead, it can no longer help the reproduction of the pathogen, nor can the host take an active part in transmitting it. High virulence is detrimental to the pathogen, and so it is bound to evolve to a lower level of virulence to better spread. In plain words, if a pathogen causes its host to lay in bed or even to die instead of happily going around and transmitting the infection, it will be outcompeted by mutants that cause less damage to the host and allows it to produce more pathogens and infect other hosts. This is the verbal description of the transmission–virulence trade-off.

In mathematical terms, the key here is that both transmission (*β*) and recovery rate (*γ*) are functions of virulence, measured here as excess death rate (*δ*). Thus, Eq. (4) can be rewritten as follows:

5 $$R_{0} \sim \;\frac{{\beta \left( \delta \right)}}{{\delta + \gamma \left( \delta \right)}} .$$

**Fig. 1:**
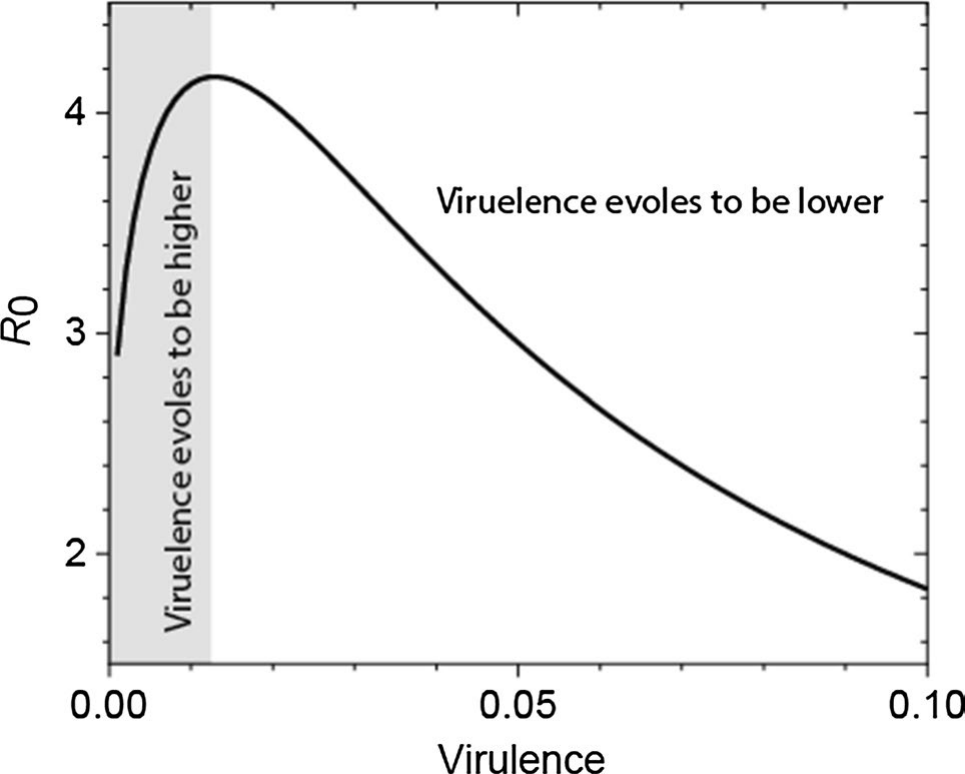
Transmission–virulence trade-off. The reproductive rate of a pathogen has a maximum as virulence increases. If initial virulence is higher than this optimal virulence, then, virulence evolves to be lower. But virulence can also increase if the initial one is lower than the optimum (the shaded area on the graph). The curve has the same shape found by Anderson and May (1982) for myxomatosis

Anderson and May (Anderson and May 1982) critiqued the original theory on exactly this ground: if these parameters are interdependent then the outcome might differ. If transmission rate is a linear function of virulence and recovery does not depend on virulence, then, both virulence and transmission rate evolve to as high as possible. This is already a counterexample to the avirulence hypothesis as it demonstrates that virulence can increase. But their main argument was that both recovery rate and transmission rate are a nonlinear, saturating function of virulence. As virulence increases, the recovery rate decreases, steeper at first (at low virulence) and tapering off at higher virulences. Thus, the benefit of a lower recovery rate, which translates to a longer infectious period, is offset by the cost of higher mortality (virulence, which shortens the infectious period). This was shown to lead to intermediate virulence (Fig. 1) backed up by data from myxomatosis (Anderson and May 1982).

The SIR model described by Eqs. 1–3 is sufficient to show the behavior the above two theories formulate. When studying the long-term the evolution of virulence, a demographics SIR model that also includes the birth of new hosts could be employed. Readers should refer to Nowak’s chapter on the mathematical virulence evolution (Nowak 2006).

The above mathematical results are based on a list of assumptions. Mathematical formulations are more exact than verbal arguments and specify the conditions under which certain statements, like ‘virulence ought to decrease,’ are valid. The formulation is based on a number of biological assumptions. Should these biological assumption change, the formulation would also change, and potentially, the outcome would change too. Here, we go through the assumptions of the avirulence and transition–virulence trade-off hypotheses, in part based on Sigmund et al. (2002) and give counterexamples and exceptions.

- *The pathogen spreads *via* contact between an infectious host and a susceptible host.* The pathogen is not spread by a vector, by water, by contaminating other objects, by attendants, or by any other means. Otherwise, infection probability is not proportional to the meeting frequency of infectious and susceptible individuals (as in Eqs. 1 and 2).
- *Infection obeys the law of mass action, so that infected and susceptible individuals meet proportional to their density in the population.* This is an assumption of the underlying epidemiological model which is rarely the case in real life. Spatial structuring of infected individuals could lead to the evolution of less virulent strains (Lion and Boots 2010, van Baalen 2002), but it is not universally true, and in some cases, limited dispersal can lead to higher virulence (Boots et al. 2004; Lion and Boots 2010).
- *Pathogens only survive in living hosts.* If the host dies, either by causes not related to the pathogen or as a consequence of the infection, the host ceases to be infectious. In Eq. (2), the deceased are removed from the infectious population. In biological terms, this means that corpses are not vehicles for further infections. But infection can be acquired from corpses, as in the case of the Ebola virus (Prescott et al. 2015) and the Nipah virus (Sazzad et al. 2013).
- *There is no coinfection with other lineages of the same pathogen.* Consequently, within the hosts, pathogens compete only with their close relatives. Within-host competition can increase virulence (Frank 1996). If virulence increases the replication rate and, through it, transmission then a more virulent lineage can be more successful even if it shortens the infectious period by killing the host.
- *Other pathogens do not interfere with the focal pathogen.* It is implied that the host either is not infected with other pathogens, or their effects can be lumped together as a constant background mortality rate. So it is assumed that other pathogens do not hamper or facilitate infection, nor is there cross-immunity present in the population. But theory and the limited amount of experimental results that are available to us suggest that coinfection affects virulence levels (Alizon et al. 2013).
- *The per-host disease-free death rate is constant.* The host does not experience increased mortality at higher age (senescence). Theory suggests that in such case the evolutionarily stable level of virulence is higher (Hamley and Koella 2021).
- *Replication of the pathogen is detrimental to the host (increases virulence) and shortens the infectious period.* There seems to be ample evidence for the pathogen’s replication being detrimental to the host (Acevedo et al. 2019). But it is also important that the increased mortality happens when the host is infectious (Acevedo et al. 2019), so the infectious period shortens (see Eq. 2). However, sometimes the detrimental effect of the infection comes after the infectious period. In such case, the higher virulence does not shorten the infectious period. An example could be when the symptoms are caused by the reaction of the host’s immune system. The severity of that reaction might be attributed to the host, and it could affect whether lower or higher virulence is evolutionary stable (Graham et al. 2005; Weiss 2002).
- *Immunity is long-lasting, and the pathogen does not evolve to escape this immunity.* If immunity wanes or the pathogen escapes immunity as it evolves, virulence can evolve differently than predicted here. For example, regular antigen escape selects for a higher virulence (Sasaki et al. 2022), as it selects for high invasion success of the new mutant.
- *The trade-off between the per-host transmission rate and the per-host disease-induced death rate conforms to a law of diminishing returns.* A recent meta-analysis of the studies on the relationship between transmission, within-host replication, and virulence (Acevedo et al. 2019) found that replication increases with virulence, and a more replicative pathogen is transmitted more easily. The leveling-off nature of these interactions is observed, but not statistically significant. The relation between transmission and virulence is roughly like required by the hypothesis but rests only on nine studies and the conclusion is not statistically significant (Acevedo et al. 2019). All in all, there is less experimental backing behind the transmission–virulence trade-off than would be desirable for a hypothesis on which a large part of our current thinking about the evolution of virulence rests.
- *The pathogen is a specialist and cannot infect other species.* If the pathogen is a generalist, it could have higher virulence in certain hosts as they cannot optimally adapt to all of their hosts (Leggett et al. 2013). Also, if the pathogen can easily jump between host species, it can begin to adapt to the new host and then, be maladapted to the original one, when it jumps back to it.

## The ambiguous experimental/observational backing of the transmission–virulence trade-off

The transmission–virulence trade-off was proposed (Anderson and May 1982) because observation on myxomatosis (see below) suggested that there might be a trade-off between these quantities. It was shown as a transmission–recovery trade-off and then, generalized, according to Eq. 5 to a trade-off between transmission and virulence. There is experimental evidence for an intermediate level of virulence being evolutionary optimal (Acevedo et al. 2019; Doumayrou et al. 2013; Jensen et al. 2006). At the same time, we know of some viruses and microbes that do not seem to cause any health-related concerns to their host. For example, sooty mangabeys (*Cercocebus atys*) do not show any symptoms of *Simian immunodeficiency virus* (SIVsmm) infection (Rey-Cuillé et al. 1998). However, this might not be solely the evolutionary achievement of the virus, but rather the host’s (Müller and De Boer 2006).

As might be evident from the list of very specific assumptions listed above, we cannot expect all pathogens to conform to the transmission–virulence trade-off. And even if some do, there is no guarantee that evolution would lead to lower virulence. Intermediate virulence only means that neither 0% virulence (avirulence) nor 100% lethality are optimal, and evolution would lead away from them (Fig. 1).

When a pathogen switches to a new host, virulence is seldom optimal. The optimal level of virulence might as well be higher or lower than the initial one. The textbook example of the evolution of decreased virulence is the case of myxomatosis in European rabbits (*Oryctolagus cuniculus*). As the initial lethality was close to 100%, myxomatosis in rabbits is bound to evolve to lower virulence (or stay the same). Myxomatosis is caused by the myxoma virus belonging to the Poxviridae family of viruses. It causes a mild disease in its original host, the Brazilian cottontail (*Sylvilagus brasiliensis*). But in the European rabbit, the myxoma virus caused a mostly fatal disease, which is why it was used to control the invasive rabbit population in Australia. The virus is carried from one rabbit to another by an arthropod vector. The virus just hijacks the arthropod, and it does not replicate in it. While vector-borne pathogens are generally out of scope for the transmission–virulence trade-off hypothesis, as myxoma virus does not replicate in the vector, the hypothesis was still applied to it. It is worth mentioning that while on average, it seems that the lethality of the Myxoma virus has lowered in the European rabbits, very virulent strains are still circulating (Kerr et al. 2015). Rabbit populations were selected to be resistant to the original strain. But as the coevolution between the new host and the pathogen progressed, instead of becoming the milder, newer variants of the virus have evolved increased immunosuppressive ability (Kerr et al. 2017).

The early history of myxomatosis is a classic case study. It is an example where evolution has led, at least initially, to lowered levels of virulence, and also its evolution is known in great detail (Kerr et al. 2015). The introduction of the virus to the Australian population was voluntary and the unfolding events were monitored and recorded. Another recent and well-documented host switch and the subsequent epidemic involves two species of *Simian immunodeficiency viruses* which became the *Human immunodeficiency virus 1* and *2* (HIVs). HIVs are retroviruses which switched hosts from other primates to humans in the early twentieth century (Sousa et al. 2017). AIDS, the disease HIV causes, was first observed as a new disease in the ‘80 s, and ever since, the evolution of the viruses is closely followed. There was an expectation that the lethality of HIV infection would decrease. This is what was found in Uganda (Blanquart et al. 2016). But virulence did not change in Switzerland (Müller et al. 2006) and increased in Italy (Müller et al. 2009), the USA (Crum-Cianflone et al. 2009; Wertheim et al. 2019), and in the Netherlands (Wymant et al. 2022). More generally, a meta-analysis found that globally CD4^+^ count of patients is decreasing quicker, viral load is increasing, thus the virus becomes more virulent (Herbeck et al. 2012).

HIV infection can be kept at bay with treatment, which raised the question of whether treatment could increase virulence. If the pathogen is in an environment with most hosts vaccinated or treated, transmission can become difficult, and only very aggressive strains might survive. The classic example of this is Marek’s disease of poultry, caused by a herpesvirus (*Marek’s disease virus*, MDV). Because of agricultural importance, a vaccine was developed for an otherwise mild disease that rarely if ever was lethal. The vaccine successfully kept symptoms at bay, but virus replication and transmission were not inhibited. Selection on limiting virulence was lifted as vaccinated birds do not suffer from the heightened virulence because of the vaccine (Read et al. 2015; Witter 1997) but can still successfully transmit the disease. Unvaccinated individuals die quicker, but the maintenance of the virus does not depend on this small fraction of the population. Not just vaccination, but also effective treatment could in principle select for higher virulence. In the case of HIV, very high coverage of antiretroviral treatment might cause an increase in virulence (Herbeck et al. 2016). But generally, we do not expect vaccination or treatment to be the cause of higher virulence (Bull and Antia 2022).

Smallpox had become more virulent over the centuries (Alcamí 2020; Mühlemann et al. 2020). There were numerous losses of immune escape genes that helped the infection to persist longer, but those genes also lessened the immunopathogenic effect of the disease. Without these genes, smallpox became the lethal disease known today and not the mild disease it was, like cowpox or chickenpox.

The evolution of a limited number of pathogens that we could either monitor from its beginning or infer its evolution from molecular data, as described above, does not seem to be in favor of pathogens becoming universally milder. Can we expect COVID-19 to become a milder disease? To answer this question, we need to go through the assumptions of the avirulence and transmission–virulence trade-off hypothesis and assess if COVID-19 conforms to them.

## COVID-19

*Severe acute respiratory syndrome-related coronavirus* SARS-CoV-2 (Gorbalenya et al. 2020) is the causative agent of COVID-19. The positive-strand RNA virus belongs to the genus *Betacoronavirus*, family Coronaviridae, and order Nidovirales.

Like the other coronaviruses infecting humans, it causes respiratory tract infection. The infection is transmitted directly from a living individual to other living individuals via droplets and aerosol. Infection through contaminated objects (fomite) plays no role in the epidemic (Onakpoya et al. 2021). After infection, an incubation period starts. The incubation period is on average 5.4 days (Xin et al. 2021), but the development of symptoms is quicker in newer variants (Wu et al. 2022). Individuals are often infectious before symptom onset (Jones et al. 2021). Some, around 17% (Byambasuren et al. 2020), do not develop symptoms; thus, the entire infection is asymptomatic. Nevertheless, asymptomatic and presymptomatic (those who develop symptoms later) individuals can be as infectious as symptomatic individuals (Jones et al. 2021; Zou et al. 2020).

As for severity, for most of the patients, the disease is a mild one (70–80% of the infected required no hospitalization even at the beginning of the pandemic (ECDC 2020, WHO 2020)). The rest requires hospital care, and a fraction of these individuals require intensive care. Severe disease develops through the interaction of the virus and the immune system of the infected. Fatal outcome happened 1–2 weeks after symptoms onset (Lefrancq et al. 2021; Linton et al. 2020; Zardini et al. 2021), albeit quicker progression of the disease was also reported (Impouma et al. 2022). Live virus shedding might be over by the time patients die.

COVID-19 fulfills some of the assumptions of the models of virulence evolution listed above.

- *It is transmitted directly from living individuals* as stated above.
- *Coinfection by different strains of SARS-CoV-2 is not widespread.* There is a possibility of coinfection with different strains of SARS-COV-2 (Francisco Jr et al. 2021; Liu et al. 2021; Rockett et al. 2022; Taghizadeh et al. 2021). The incidence of such coinfection is around 5 (Liu et al. 2021) to 13% (Taghizadeh et al. 2021). There is one report suggesting longer virus shedding and more severe disease in those coinfected with two variants (Pedro et al. 2021).
- *Coinfection with other pathogens does not interfere significantly.* While there are some indications that patients can be infected by other pathogens besides SARS-CoV-2 (Jiang et al. 2020), it is not typical (Calcagno et al. 2021). We can assume that pathogen interference plays an insignificant role in the current evolutionary trends of SARS-CoV-2. But even without coinfection, pathogens can have a strong indirect effect on each other. The non-pharmaceutical intervention during the COVID-19 pandemic also prevented infection by other respiratory viruses (Kim et al. 2021). A lineage of influenza B virus (B/Yamagata/16/1988) might have gone extinct (Koutsakos et al. 2021). In the fall/winter of 2022, the circulation of respiratory syncytial virus (RSV) was intensified (European Center for Disease Prevention and Control 2022; Munkstrup et al. 2023). While we do not know for sure if it is connected to the COVID-19 pandemic, the new pathogen and the preventive measures of first two years of the pandemic might have caused a change the known rithms of seasonal epidemics. The long-term evolution of SARS-CoV2 can be affected by the other seasonal respiratory pathogens.

However, other assumptions are violated.

- *A higher viral load does not translate to a higher risk of death.* The data on this is ambiguous (Dadras et al. 2022), and while in some cases scientists found a positive correlation between viral load and disease severity, in others, there was no correlation or negative correlation.
- *Immunity is not long-lasting, and the pathogen evolves to escape immunity.* There is waning immunity to SARS-CoV2 irrespective whether it came from former infection or vaccination (Gazit et al. 2022; Goldberg et al. 2022; Thomas et al. 2021; Vokó et al. 2022). Prior infection offers a limited cross-immunity to other variants (Suryawanshi et al. 2022; Tang et al. 2022). These taken together can select for a higher virulence (Sasaki et al. 2022) as opposed to a lower one.
- *Humans are not the only possible host for SARS-CoV-2.* While humans are the main host of SARS-CoV-2, other species, such as mink (Devaux et al. 2021), white-tailed deer (Mallapaty 2022), or cats (Doliff and Martens 2022; Halfmann et al. 2020) can catch the virus which can spread among them. Not only can these spill-over infections act as reservoirs of SARS-CoV-2, but the virus may evolve in non-human hosts and may later reinfect humans. We cannot forecast the effect of these evolved pathogen on humans.

## The evolution of SARS-CoV-2

The assumptions of the main evolutionary theories on virulence evolution are not met. Consequently, we cannot invoke their prediction when it comes to the evolution of the virulence of SARS-CoV-2 infection. We can expect the virus to become endemic (King 2021) like four of the coronaviruses known to infect humans, but we cannot expect it to become harmless (Katzourakis 2022).

Antia and coworkers (Lavine et al. 2021) have argued that COVID-19 can become a seasonal infection that causes very few deaths. Their argument is heavily based on two factors: (1) mortality is high only at old age, and (2) immunity is long-lasting. The first assumption fits perfectly. The population is then infected at a young age, where the risk of death by COVID-19 is low. If there would be lifelong sterilizing immunity, then, the virus could not circulate (like *varicella*). But as reinfection is possible and former infections protect from severe disease, then, on a population level, COVID-19 will become a mild, seasonal infectious disease (like influenza).

In the case of COVID-19, the problem is that immunity does not last long. We still do not yet know if cross-immunity from a former infection protects against severe disease caused by a new variant (so far vaccination does offer protection from severe disease Altmann and Boyton 2022; Scott et al. 2021)). Moreover, most virus transmission occurs well before the disease progresses to a severe one. This lessens the selection pressure on a lower virulence (Day et al. 2020). The predicted evolution toward lower virulence depends on a higher death rate shortening the infectious period (see Eqs. (4) and (5)). This is not the case with COVID-19, which prompted Katzourakis (2022) and Miller and Metcalf (2022) to point out that we cannot apply the transmission–virulence trade-off and thus, cannot expect the disease to become milder because of it.

In fact, some evidence suggests that COVID-19 has become more lethal and more transmissible at the same time. The original variant detected in Wuhan in 2020 had a crude fatality ratio of 0.7% (higher at the very beginning of the epidemic) and a basic reproduction number (*R*_0_) of 2–2.5 (WHO 2020) (later estimates are in the range of 1.4–6.49, with a median of 2.79 (Liu et al. 2020)). The basic reproduction number is the mean number of secondary infections generated by an infected individual in a susceptible population. As the population was susceptible to the novel pathogen, *R*_0_ could be estimated. For later variants, they compared their transmissibility to the original variants (the new transmissibilities are always higher for variants of concern and variants of interest (Campbell et al. 2021)). A novel mutation from aspartic acid to glycine on the 614 position of the spike protein (D614G) rose to prominence and replaced the original variant by the summer of 2020 (Korber et al. 2020; Martin et al. 2021; Tao et al. 2021). The D614G variant has an increased transmission rate (Korber et al. 2020, Volz et al. 2021a), as it has a higher affinity to the ACE2 receptors. The estimated *R*_0_ is around 3–4.5 (Leung et al. 2021), a roughly 30% increase from the original variant. The lethality of this variant is the same (Volz et al. 2021a). At the end of 2020, a number of more infectious variants emerged. In Europe, first the Alpha (B1.1.17) variant rose to prominence in 2021 (Tao et al. 2021). The lethality of this variant is about 50% higher than that of the D614G variant (Challen et al. 2021, Davies et al. 2021b, Grint et al. 2021). Transmissibility of the Alpha variant is 50–100% higher (Davies et al. 2021a, Volz et al. 2021b). Then, the Alpha variant was replaced with the Delta (B.1.617.2) variant. So, it has even higher transmissibility, with estimated* R*_0_ = 5.08 (Liu and Rocklöv 2021). Delta might be as lethal as Alpha (Zhao et al. 2022). In 2022, the Omicron (B.1.1.529) variant replaced Delta as the dominant variant. Interestingly, while it has higher transmissibility (*R*_0_ = 8.2 (Liu and Rocklöv 2022)), it has a lower associated risk of death (Nyberg et al. 2022). Omicron broke a trend of increased transmissibility coupled with increased or similar lethality. Other notable variants also mostly conform to the higher transmissibility and higher mortality rate pattern. For example, the Beta (B.1.351) variant, which was first detected in South Africa, has a higher transmissibility (Tegally et al. 2021) and a higher mortality than the original variant (Funk et al. 2021). Similarly, the Gamma (P.1) variant, which was first detected in Brazil, has a higher transmissibility and a higher mortality rate (Faria et al. 2021; Funk et al. 2021). Markov and coworkers (2022) warn us that the “lower severity of Omicron is nothing but a lucky coincidence,” and we should expect more transmissible and more immune evasive variants to emerge (see also (Otto et al. 2021)).

Virulence is a trait that is influenced by both the pathogen and the host (Read 1994). Mostly we discuss virulence as a trait exclusively of the pathogen. But various traits of the host affects the severity and lethality of the disease (for COIVD-19 see the exhaustive review by Zsichla and Müller (2023)). Furthermore, the host is also selected to decrease virulence. While the generation time of humans is vastly longer than the generation time of a virus (measured as the time between infecting new hosts), there are other ways to counter the effect of a pathogen. Proper health care, antivirals (if available), and vaccination are cultural advances that help us lower the mortality/morbidity caused by a viral pathogen. Vaccines, because of the example of Marek’s disease of poultry, are feared to select for higher virulence, but this is very unlikely (Bull and Antia 2022; Miller and Metcalf 2022). Unlike the vaccine against Marek’s disease, the vaccines available against SARS-CoV-2 also lowers transmission (albeit does not eliminate it) (Braeye et al. 2021; Eyre et al. 2022; Pritchard et al. 2021; Tan et al. 2023).

Consider for a moment that SARS-CoV-2/COVID-19 fulfills the assumptions of the transmission–virulence trade-off. Even then, evolution is not guaranteed to arrive at the optimal trait value anytime soon or ever. Even if there is a combination of traits that would lead to decreased virulence, it might not be evolutionary reachable or maintainable. To consider a trait (or combination of traits), an endpoint of evolution (in an otherwise constant environment), it needs to be locally evolutionary stable and should be reachable from its genetic vicinity (Dieckmann 2002; Geritz et al. 1998; Meszéna et al. 2002). Any optimal trait should first be reached in genotype space. Due to the vastness of genotype space, only a small fraction of it can be explored. This fraction lies on the smoother part of the fitness landscape (i.e., on those parts, where small genetic changes result in small changes in fitness). If a fitness peak is surrounded by a deep fitness chasm, it will never be reached. The notion of *convergence stability* means that once an evolving population reaches the vicinity of the fitness peak, it can and will climb it. *Evolutionary stability* means that no rare mutant can invade a population having this trait (Maynard Smith 1982). When analyzing complex fitness landscapes, we might narrow our focus to local mutations. Consequently, an evolutionary stable trait is a local fitness peak in the landscape. We do not consider very large jumps in genotype space (changes can still be considerable in phenotype space). So-called Garden of Eden fixed strategies are such that they are evolutionary stable, but not convergence-stable, so they cannot be reached (Geritz et al. 1998). There are examples of such dynamics in the theoretical epidemiological literature (Ashby and King 2017; Best et al. 2013, 2014). The take-home message of evolutionary theory in this regard is that the global optimum might not be reachable for various reasons.

Alizon and Sofonea (2021) rightfully point out that a pathogen that freshly changed host is maladapted to it, and we should not expect it to lie on some smooth fitness trajectory to optimality. On the contrary, we should expect that reaching the optimum is difficult and could involve virulence levels that are way different from the final evolutionary optimum (Bull and Ebert 2008). Furthermore, epistasis and pleiotropic effect can also complicate the expected smooth evolution. Epistasis means that a mutation could have a different effect based on the genetic background. The N501Y mutation found in the spike protein of the Alpha and latter variants increases affinity to the ACE2 receptor, thus increasing transmissibility (Liu et al. 2022). The mutation to glycine on the 446 position helps escape antibodies but incurs a cost in the affinity. The affinity drop is less in the original variant, but more pronounced with the N501Y mutation (Starr et al. 2022). So, with the N501Y mutation background, this mutation would not have risen to prominence. This observation is also an example of a pleiotropic effect: The mutation has an immunoevasive effect and lowers the affinity of the spike protein to the receptor. Furthermore, while one might think that it is mostly transmission or immune evasion that shape the fitness landscape of SARS-CoV-2, mutations elsewhere in its genome also significantly contribute to it (Obermeyer et al. 2022): six of the top 20 most influential mutation are not associated with the spike protein. Such convoluted interactions between mutations and traits seem to be common which makes short-term predictions on the evolution of any pathogen especially hard.

What we can safely say is that the lethality (virulence) of SARS-CoV2 will decrease, increase, or stay the same.

## Conclusion for future biology

The maturity of a field in natural science can be seen in the number of predictive mathematical formulas employed. It is high in physics, moderate in chemistry, and very few in biology. Biology is still in its juvenile stage. New data are generated faster than we can make sense of it. Still, data are not always generated on what we need but what is available and easy to do. Pathogen management should have a sound theoretical basis (Dieckmann et al. 2002). Theory, in this field, is far ahead of observations and experiments required to test said theories. We just do not yet know, which theory applies to which disease. For that, we need more targeted experiments. At the same time, theoretical papers should always incorporate what we already know about pathogens and the diseases they cause (Alizon et al. 2013; Cressler et al. 2015).

## References

[CR1] Acevedo MA, Dillemuth FP, Flick AJ, Faldyn MJ, Elderd BD (2019). Virulence-driven trade-offs in disease transmission: a meta-analysis. Evolution.

[CR2] Alcamí A (2020). Was smallpox a widespread mild disease?. Science.

[CR3] Alizon S, Michalakis Y (2015). Adaptive virulence evolution: the good old fitness-based approach. Trends Ecol Evol.

[CR4] Alizon S, Sofonea MT (2021). SARS-CoV-2 virulence evolution: Avirulence theory, immunity and trade-offs. J Evol Biol.

[CR5] Alizon S, de Roode JC, Michalakis Y (2013). Multiple infections and the evolution of virulence. Ecol Lett.

[CR6] Altmann DM, Boyton RJ (2022). COVID-19 vaccination: The road ahead. Science.

[CR7] Anderson RM, May RM (1982). Coevolution of hosts and parasites. Parasitology.

[CR8] Ashby B, King KC (2017). Friendly foes: The evolution of host protection by a parasite. Evolut Lett.

[CR9] Best A, Tidbury H, White A, Boots M (2013). The evolutionary dynamics of within-generation immune priming in invertebrate hosts. J R Soc Interface.

[CR10] Best A, White A, Boots M (2014). The coevolutionary implications of host tolerance. Evolution.

[CR11] Blanquart F, Grabowski MK, Herbeck J, Nalugoda F, Serwadda D, Eller MA (2016). A transmission-virulence evolutionary trade-off explains attenuation of HIV-1 in Uganda. Elife.

[CR12] Boots M, Hudson PJ, Sasaki A (2004). Large shifts in pathogen virulence relate to host population structure. Science.

[CR13] Braeye T, Cornelissen L, Catteau L, Haarhuis F, Proesmans K, De Ridder K, a. (2021). Vaccine effectiveness against infection and onwards transmission of COVID-19: Analysis of Belgian contact tracing data, January-June 2021. Vaccine.

[CR14] Bull JJ, Antia R (2022). Which ‘imperfect vaccines’ encourage the evolution of higher virulence?. Evolut, Med, Public Health.

[CR15] Bull JJ, Ebert D (2008). Invasion thresholds and the evolution of nonequilibrium virulence. Evolut Appl.

[CR16] Byambasuren O, Cardona M, Bell K, Clark J, McLaws M-L, Glasziou P (2020). Estimating the extent of asymptomatic COVID-19 and its potential for community transmission: Systematic review and meta-analysis. Off J Assoc Med Microbiol Infect Disease Canada.

[CR17] Calcagno A, Ghisetti V, Burdino E, Trunfio M, Allice T, Boglione L (2021). Co-infection with other respiratory pathogens in COVID-19 patients. Clin Microbiol Infect.

[CR18] Campbell F, Archer B, Laurenson-Schafer H, Jinnai Y, Konings F, Batra N (2021). Increased transmissibility and global spread of SARS-CoV-2 variants of concern as at June 2021. Eurosurveillance.

[CR19] CDC (2020) disease burden of influenza. Vol. 2020, https://www.cdc.gov/flu/about/burden/index.html.

[CR20] Challen R, Brooks-Pollock E, Read JM, Dyson L, Tsaneva-Atanasova K, Danon L (2021). Risk of mortality in patients infected with SARS-CoV-2 variant of concern 202012/1: matched cohort study. BMJ.

[CR21] Cressler CE, McLeod DV, Rozins C, Van Den Hoogen J, Day T (2015). The adaptive evolution of virulence: a review of theoretical predictions and empirical tests. Parasitology.

[CR22] Crum-Cianflone N, Eberly L, Zhang Y, Ganesan A, Weintrob A, Marconi V (2009). Is HIV becoming more virulent? Initial CD4 cell counts among HIV seroconverters during the course of the HIV epidemic: 1985–2007. Clin Infect Dis.

[CR23] Dadras O, Afsahi AM, Pashaei Z, Mojdeganlou H, Karimi A, Habibi P (2022). The relationship between COVID-19 viral load and disease severity: a systematic review. Immun, Inflam Dis.

[CR24] Davies NG, Abbott S, Barnard RC, Jarvis CI, Kucharski AJ, Munday JD (2021). Estimated transmissibility and impact of SARS-CoV-2 lineage B.1.1.7 in England. Science.

[CR25] Davies NG, Jarvis CI, van Zandvoort K, Clifford S, Sun FY, Funk S (2021). Increased mortality in community-tested cases of SARS-CoV-2 lineage B.1.1.7. Nature.

[CR26] Day T, Gandon S, Lion S, Otto SP (2020). On the evolutionary epidemiology of SARS-CoV-2. Curr Biol.

[CR27] Devaux CA, Pinault L, Delerce J, Raoult D, Levasseur A, Frutos R (2021). Spread of mink SARS-CoV-2 variants in Humans: a model of sarbecovirus interspecies evolution. Front Microbiol.

[CR29] Dieckmann U, Dieckmann U (2002). Adaptive dynamics of pathogen-host iteraction. Adaptive dynamics of infectious diseases.

[CR28] Dieckmann U, Metz JAJ, Sabelis MW, Sigmund K (2002). Adaptive dynamics of infectious diseases.

[CR30] Doliff R, Martens P (2022). Cats and SARS-CoV-2: a scoping review. Animals.

[CR31] Doumayrou J, Avellan A, Froissart R, Michalakis Y (2013). An experimental test of the transmission-virulence trade-off hypothesis in a plant virus. Evolution.

[CR32] ECDC (2020) Coronavirus disease 2019 (COVID-19) in the EU/EEA and the UK – eighth update. ECDC.

[CR33] European Centre for Disease Prevention and Control (2022) Intensified circulation of respiratory syncytial virus (RSV) and associated hospital burden in the EU/EEA. ECDC, Stockholm.

[CR34] Eyre DW, Taylor D, Purver M, Chapman D, Fowler T, Pouwels KB (2022). Effect of Covid-19 vaccination on transmission of Alpha and Delta bariants. New Engl J Med.

[CR35] Faria NR, Mellan TA, Whittaker C, Claro IM, Candido DdS, Mishra S (2021). Genomics and epidemiology of the P.1 SARS-CoV-2 lineage in Manaus Brazil. Science.

[CR36] Francisco Jr RdS, Benites LF, Lamarca AP, de Almeida LGP, Hansen AW, Gularte JS (2021). Pervasive transmission of E484K and emergence of VUI-NP13L with evidence of SARS-CoV-2 co-infection events by two different lineages in Rio Grande do Sul. Brazil. Virus Res.

[CR37] Frank SA (1996). Models of parasite virulence. Q Rev Biol.

[CR38] Funk T, Pharris A, Spiteri G, Bundle N, Melidou A, Carr M (2021). Characteristics of SARS-CoV-2 variants of concern B.1.1.7, B.1.351 or P.1: data from seven EU/EEA countries, weeks 38/2020 to 10/2021. Eurosurveillance.

[CR39] Gazit S, Shlezinger R, Perez G, Lotan R, Peretz A, Ben-Tov A (2022). Severe acute respiratory syndrome coronavirus 2 (SARS-CoV-2) naturally acquired immunity versus vaccine-induced immunity, reinfections versus breakthrough infections: A retrospective cohort study. Clin Infect Dis.

[CR40] Geoghegan JL, Holmes EC (2018). The phylogenomics of evolving virus virulence. Nat Rev Genet.

[CR41] Geritz SAH, Kisdi É, Meszéna G, Metz JAJ (1998). Evolutionarily singular strategies and the adaptive growth and branching of the evolutionary tree. Evol Ecol.

[CR42] Goldberg Y, Mandel M, Bar-On YM, Bodenheimer O, Freedman LS, Ash N (2022). Protection and waning of natural and hybrid immunity to SARS-CoV-2. New Engl J Med.

[CR43] Gorbalenya AE, Baker SC, Baric RS, de Groot RJ, Drosten C, Gulyaeva AA (2020). The species Severe acute respiratory syndrome-related coronavirus: classifying 2019-nCoV and naming it SARS-CoV-2. Nat Microbiol.

[CR44] Graham AL, Allen JE, Read AF (2005). Evolutionary causes and consequences of immunopathology. Annu Rev Ecol Evol Syst.

[CR45] Grint DJ, Wing K, Williamson E, McDonald HI, Bhaskaran K, Evans D (2021). Case fatality risk of the SARS-CoV-2 variant of concern B.1.1.7 in England, 16 November to 5 February. Eurosurveillance.

[CR46] Halfmann PJ, Hatta M, Chiba S, Maemura T, Fan S, Takeda M (2020). Transmission of SARS-CoV-2 in domestic cats. New Engl J Med.

[CR47] Hamley JID, Koella JC (2021). Parasite evolution in an age-structured population. J Theor Biol.

[CR48] Herbeck JT, Müller V, Maust BS, Ledergerber B, Torti C, Di Giambenedetto S (2012). Is the virulence of HIV changing? A meta-analysis of trends in prognostic markers of HIV disease progression and transmission. AIDS.

[CR49] Herbeck JT, Mittler JE, Gottlieb GS, Goodreau SM, Murphy JT, Cori A (2016). Evolution of HIV virulence in response to widespread scale up of antiretroviral therapy: a modeling study. Virus Evolution.

[CR50] Impouma B, Carr ALJ, Spina A, Mboussou F, Ogundiran O, Moussana F (2022). Time to death and risk factors associated with mortality among COVID-19 cases in countries within the WHO African region in the early stages of the COVID-19 pandemic. Epidemiol Infect.

[CR51] Jensen KH, Little TJ, Skorping A, Ebert D (2006). Empirical support for optimal virulence in a castrating parasite. PLoS Biol.

[CR52] Jiang S, Liu P, Xiong G, Yang Z, Wang M, Li Y, Yu X-j (2020). Coinfection of SARS-CoV-2 and multiple respiratory pathogens in children. Clin Chem Lab Med (CCLM).

[CR53] Jones TC, Biele G, Mühlemann B, Veith T, Schneider J, Beheim-Schwarzbach J (2021). Estimating infectiousness throughout SARS-CoV-2 infection course. Science.

[CR54] Katzourakis A (2022). COVID-19: endemic doesn’t mean harmless. Nature.

[CR55] Kermack WO, McKendrick AG, Walker GT (1927). A contribution to the mathematical theory of epidemics. Proc R Soc Lond Ser a, Contain Pap Math Phys Character.

[CR56] Kerr PJ, Liu J, Cattadori I, Ghedin E, Read AF, Holmes EC (2015). Myxoma virus and the Leporipoxviruses: an evolutionary paradigm. Viruses.

[CR57] Kerr PJ, Cattadori IM, Liu J, Sim DG, Dodds JW, Brooks JW (2017). Next step in the ongoing arms race between myxoma virus and wild rabbits in Australia is a novel disease phenotype. PNAS.

[CR58] Kim J-H, Roh YH, Ahn JG, Kim MY, Huh K, Jung J, Kang J-M (2021). Respiratory syncytial virus and influenza epidemics disappearance in Korea during the 2020–2021 season of COVID-19. Int J Infect Dis.

[CR59] King A (2021). The coronavirus could end up mild like a common cold. New Sci.

[CR60] Korber B, Fischer WM, Gnanakaran S, Yoon H, Theiler J, Abfalterer W, Montefiori DC (2020). Tracking changes in SARS-CoV-2 spike: evidence that D614G increases infectivity of the COVID-19 virus. Cell.

[CR61] Koutsakos M, Wheatley AK, Laurie K, Kent SJ, Rockman S (2021). Influenza lineage extinction during the COVID-19 pandemic?. Nat Rev Microbiol.

[CR62] Lavine JS, Bjornstad ON, Antia R (2021). Immunological characteristics govern the transition of COVID-19 to endemicity. Science.

[CR63] Lefrancq N, Paireau J, Hozé N, Courtejoie N, Yazdanpanah Y, Bouadma L (2021). Evolution of outcomes for patients hospitalised during the first 9 months of the SARS-CoV-2 pandemic in France: a retrospective national surveillance data analysis. Lancet Reg Health Euro.

[CR64] Leggett HC, Buckling A, Long GH, Boots M (2013). Generalism and the evolution of parasite virulence. Trends Ecol Evol.

[CR65] Leung K, Pei Y, Leung GM, Lam TT, Wu JT (2021). Estimating the transmission advantage of the D614G mutant strain of SARS-CoV-2, December 2019 to June 2020. Eurosurveillance.

[CR66] Linton NM, Kobayashi T, Yang Y, Hayashi K, Akhmetzhanov AR, Jung S-m (2020). Incubation period and other epidemiological characteristics of 2019 novel coronavirus infections with right truncation: a statistical analysis of publicly available case data. J Clin Med.

[CR67] Lion S, Boots M (2010). Are parasites ‘‘prudent’’ in space?. Ecol Lett.

[CR68] Liu Y, Rocklöv J (2021). The reproductive number of the Delta variant of SARS-CoV-2 is far higher compared to the ancestral SARS-CoV-2 virus. J Travel Med.

[CR69] Liu Y, Rocklöv J (2022). The effective reproductive number of the Omicron variant of SARS-CoV-2 is several times relative to Delta. J Travel Med.

[CR70] Liu Y, Gayle AA, Wilder-Smith A, Rocklöv J (2020). The reproductive number of COVID-19 is higher compared to SARS coronavirus. J Travel Med.

[CR71] Liu R, Wu P, Ogrodzki P, Mahmoud S, Liang K, Liu P (2021). Genomic epidemiology of SARS-CoV-2 in the UAE reveals novel virus mutation, patterns of co-infection and tissue specific host immune response. Sci Rep.

[CR72] Liu Y, Liu J, Plante KS, Plante JA, Xie X, Zhang X (2022). The N501Y spike substitution enhances SARS-CoV-2 infection and transmission. Nature.

[CR73] Mallapaty S (2022). COVID is spreading in deer. What does that mean for the pandemic?. Nature.

[CR74] Markov PV, Katzourakis A, Stilianakis NI (2022). Antigenic evolution will lead to new SARS-CoV-2 variants with unpredictable severity. Nat Rev Microbiol.

[CR75] Martin DP, Weaver S, Tegally H, San JE, Shank SD, Wilkinson E (2021). The emergence and ongoing convergent evolution of the SARS-CoV-2 N501Y lineages. Cell.

[CR76] Maynard Smith J (1982). Evolution and the Theory of Games.

[CR77] Meszéna G, Kisdi É, Dieckmann U, Geritz SAH, Metz JAJ (2002). Evolutionary optimisation models and matrix games in the unified perspective of adaptive dynamics. Selection.

[CR78] Méthot PO (2012). Why do parasites harm their host? On the origin and legacy of Theobald Smith's "law of declining virulence" – 1900–1980. Hist Philos Life Sci.

[CR79] Miller IF, Metcalf CJE (2022). Assessing the risk of vaccine-driven virulence evolution in SARS-CoV-2. R Soc Open Sci.

[CR80] Mühlemann B, Vinner L, Margaryan A, Wilhelmson H, de la Fuente CC, Allentoft ME (2020). Diverse variola virus (smallpox) strains were widespread in northern Europe in the Viking Age. Science.

[CR81] Müller V, Maggiolo F, Suter F, Ladisa N, De Luca A, Antinori A (2009). Increasing clinical virulence in two decades of the Italian HIV epidemic. PLoS Path.

[CR82] Müller V and De Boer RJ (2006) The integration hypothesis: An evolutionary pathway to benign SIV infection. PLoS Path, 2, e15, doi: 10.1371/journal.ppat.0020015 10.1371/journal.ppat.0020015 PMC1434788 16609728

[CR83] Viktor Müller, Bruno Ledergerber, Luc Perrin, Thomas Klimkait, Hansjakob Furrer, Amalio Telenti, Enos Bernasconi, Pietro Vernazza, Huldrych F Günthard, Sebastian Bonhoeffer (2006) Stable virulence levels in the HIV epidemic of Switzerland over two decades. AIDS, 20, 889–89410.1097/01.aids.0000218553.51908.6b 10.1097/01.aids.0000218553.51908.6b 16549973

[CR84] Munkstrup C, Lomholt FK, Emborg H-D, Møller KL, Krog JS, Trebbien R, Vestergaard LS (2023). Early and intense epidemic of respiratory syncytial virus (RSV) in Denmark, August to December 2022. Eurosurveillance.

[CR85] Nowak MA (2006). Evolution of virulence.

[CR86] Nyberg T, Ferguson NM, Nash SG, Webster HH, Flaxman S, Andrews N (2022). Comparative analysis of the risks of hospitalisation and death associated with SARS-CoV-2 omicron (B.1.1.529) and delta (B.1.617.2) variants in England: a cohort study. The Lancet.

[CR87] Obermeyer F, Jankowiak M, Barkas N, Schaffner SF, Pyle JD, Yurkovetskiy L (2022). Analysis of 6.4 million SARS-CoV-2 genomes identifies mutations associated with fitness. Science.

[CR88] Onakpoya IJ, Heneghan CJ, Spencer EA, Brassey J, Plüddemann A, Evans DH (2021). SARS-CoV-2 and the role of fomite transmission: a systematic review. F1000Research.

[CR89] Otto SP, Day T, Arino J, Colijn C, Dushoff J, Li M (2021). The origins and potential future of SARS-CoV-2 variants of concern in the evolving COVID-19 pandemic. Curr Biol.

[CR90] Paget J, Spreeuwenberg P, Charu V, Taylor RJ, Iuliano AD, Bresee J (2019). Global mortality associated with seasonal influenza epidemics: new burden estimates and predictors from the GLaMOR Project. J Glob Health.

[CR91] Pedro N, Silva CN, Magalhães AC, Cavadas B, Rocha AM, Moreira AC (2021). Dynamics of a dual SARS-CoV-2 lineage co-infection on a prolonged viral shedding COVID-19 case: insights into clinical severity and disease duration. Microorganisms.

[CR92] Phelps EA, Sharot T (2008). How (and why) emotion enhances the subjective sense of recollection. Curr Dir Psychol Sci.

[CR93] Prescott J, Bushmaker T, Fischer R, Miazgowicz K, Judson S, Munster VJ (2015). Postmortem stability of Ebola virus. Emerging Infect Dis.

[CR94] Pritchard E, Matthews PC, Stoesser N, Eyre DW, Gethings O, Vihta K-D (2021). Impact of vaccination on new SARS-CoV-2 infections in the United Kingdom. Nat Med.

[CR95] Read AF (1994). The evolution of virulence. Trends Microbiol.

[CR96] Read AF, Baigent SJ, Powers C, Kgosana LB, Blackwell L, Smith LP (2015). Imperfect vaccination can enhance the transmission of highly virulent pathogens. PLoS Biol.

[CR97] Rey-Cuillé MA, Berthier JL, Bomsel-Demontoy MC, Chaduc Y, Montagnier L, Hovanessian AG, Chakrabarti LA (1998). Simian immunodeficiency virus replicates to high levels in sooty mangabeys without inducing disease. J Virol.

[CR98] Rockett RJ, Draper J, Gall M, Sim EM, Arnott A, Agius JE (2022). Co-infection with SARS-CoV-2 Omicron and Delta variants revealed by genomic surveillance. Nat Comm.

[CR99] Sasaki A, Lion S, Boots M (2022). Antigenic escape selects for the evolution of higher pathogen transmission and virulence. Nat Ecol Evol.

[CR100] Sazzad HM, Hossain MJ, Gurley ES, Ameen KM, Parveen S, Islam MS (2013). Nipah virus infection outbreak with nosocomial and corpse-to-human transmission, Bangladesh. Emerg Infect Dis.

[CR101] Scott J, Richterman A, Cevik M (2021). Covid-19 vaccination: evidence of waning immunity is overstated. BMJ.

[CR102] Sigmund K, Sabelis MW, Dieckmann U, Metz JAJ, Dieckmann U (2002). Introduction. Adaptive Dynamics of Infectious Diseases.

[CR103] Smith T (1904). Some problems in the life history of pathogenic microorganisms. Science.

[CR104] Sousa JD, Müller V, Vandamme A-M (2017). The epidemic emergence of HIV: what novel enabling factors were involved?. Futur Virol.

[CR105] Starr TN, Greaney AJ, Hannon WW, Loes AN, Hauser K, Dillen JR (2022). Shifting mutational constraints in the SARS-CoV-2 receptor-binding domain during viral evolution. Science.

[CR106] Suryawanshi RK, Chen IP, Ma T, Syed AM, Brazer N, Saldhi P (2022). Limited cross-variant immunity from SARS-CoV-2 Omicron without vaccination. Nature.

[CR107] Taghizadeh P, Salehi S, Heshmati A, Houshmand SM, InanlooRahatloo K, Mahjoubi F (2021). Study on SARS-CoV-2 strains in Iran reveals potential contribution of co-infection with and recombination between different strains to the emergence of new strains. Virology.

[CR108] Talarico JM, Rubin DC (2003). Confidence, not consistency, characterizes flashbulb memories. Psychol Sci.

[CR109] Tan ST, Kwan AT, Rodríguez-Barraquer I, Singer BJ, Park HJ, Lewnard JA (2023). Infectiousness of SARS-CoV-2 breakthrough infections and reinfections during the Omicron wave. Nat Med.

[CR110] Tang J, Novak T, Hecker J, Grubbs G, Zahra FT, Bellusci L (2022). Cross-reactive immunity against the SARS-CoV-2 Omicron variant is low in pediatric patients with prior COVID-19 or MIS-C. Nat Comm.

[CR111] Tao K, Tzou PL, Nouhin J, Gupta RK, de Oliveira T, Kosakovsky Pond SL (2021). The biological and clinical significance of emerging SARS-CoV-2 variants. Nat Rev Genet.

[CR112] Tegally H, Wilkinson E, Giovanetti M, Iranzadeh A, Fonseca V, Giandhari J (2021). Detection of a SARS-CoV-2 variant of concern in South Africa. Nature.

[CR113] Thomas SJ, Moreira ED, Kitchin N, Absalon J, Gurtman A, Lockhart S (2021). Safety and efficacy of the BNT162b2 mRNA Covid-19 vaccine through 6 months. New Engl J Med.

[CR114] van Baalen M, Dieckmann U (2002). Contact networks and the evolution of virulence. Adaptive Dynamics of Infectious Diseases.

[CR115] Vokó Z, Kiss Z, Surján G, Surján O, Barcza Z, Wittmann I (2022). Effectiveness and waning of protection with different SARS-CoV-2 primary and booster vaccines during the Delta pandemic wave in 2021 in Hungary (HUN-VE 3 Study). Front Immunol.

[CR116] Volz E, Mishra S, Chand M, Barrett JC, Johnson R, Geidelberg L, Hinsley WR, Laydon DJ, Dabrera G, O’Toole Á, Amato R, Ragonnet-Cronin M, Harrison I, Jackson B, Ariani CV, Boyd O, Loman NJ, McCrone JT, Gonçalves S, Jorgensen D, Myers R, Hill V, Jackson DK, Gaythorpe K, Groves N, Sillitoe J, Kwiatkowski DP, Flaxman S, Ratmann O, Bhatt S, Hopkins S, Gandy A, Rambaut A, Ferguson NM (2021). Assessing transmissibility of SARS-CoV-2 lineage B.1.1.7 in England. Nature.

[CR117] Volz E, Hill V, McCrone JT, Price A, Jorgensen D, O’Toole Á (2021). Evaluating the effects of SARS-CoV-2 spike mutation D614G on transmissibility and pathogenicity. Cell.

[CR118] Weiss RA (2002). Virulence and pathogenesis. Trends Microbiol.

[CR119] Wertheim JO, Oster AM, Switzer WM, Zhang C, Panneer N, Campbell E (2019). Natural selection favoring more transmissible HIV detected in United States molecular transmission network. Nat Comm.

[CR120] WHO (2020) Report of the WHO-china joint mission on coronavirus disease 2019 (COVID-19). WHO.

[CR121] Witter RL (1997). Increased virulence of Marek's disease virus field isolates. Avian Dis.

[CR122] Wu Y, Kang L, Guo Z, Liu J, Liu M, Liang W (2022). Incubation period of COVID-19 caused by unique SARS-CoV-2 strains: a systematic review and meta-analysis. JAMA Netw Open.

[CR123] Wymant C, Bezemer D, Blanquart F, Ferretti L, Gall A, Hall M (2022). A highly virulent variant of HIV-1 circulating in the Netherlands. Science.

[CR124] Xin H, Wong JY, Murphy C, Yeung A, Taslim Ali S, Wu P, Cowling BJ (2021). The incubation period distribution of coronavirus disease 2019: a systematic review and meta-analysis. Clin Infect Dis.

[CR125] Zardini A, Galli M, Tirani M, Cereda D, Manica M, Trentini F, Merler S (2021). A quantitative assessment of epidemiological parameters required to investigate COVID-19 burden. Epidemics.

[CR126] Zhao S, Lou J, Cao L, Chong KC, Zee BCY, Chan PKS, Wang MH (2022). Differences in the case fatality risks associated with SARS-CoV-2 Delta and non-Delta variants in relation to vaccine coverage: an early ecological study in the United Kingdom Infect. Genet Evol.

[CR127] Zou L, Ruan F, Huang M, Liang L, Huang H, Hong Z, Jianxiang Y, Kang M, Song Y, Xia J, Guo Q, Song T, He J, Yen H-L, Peiris M, Jie W (2020). SARS-CoV-2 viral load in upper respiratory specimens of infected patients. New Engl J Med.

[CR128] Zsichla L, Müller V (2023). Risk factors of severe COVID-19: a review of host, viral and environmental factors. Viruses.

